# Variation Between LGBT Estimates and State Policy Context

**DOI:** 10.1007/s11113-025-09938-2

**Published:** 2025-01-28

**Authors:** Lee A. Brady, Christopher A. Julian, Wendy D. Manning

**Affiliations:** Department of Sociology, Center for Family and Demographic Research, Bowling Green State University, Bowling Green, OH 43403 USA

**Keywords:** State policy, Sexual minorities, Structural stigma, Minority stress

## Abstract

State-level social policy and LGBT (lesbian, gay, bisexual, and transgender) population concentration are key measures that are often used as indicators reflecting geographic social climate. Still, research has yet to investigate how they may be interrelated, including the degree to which the LGBT population are subject to certain policies. Using population-based experimental data from the Household Pulse Survey and policy measures from the Movement Advancement Project, we compared measures of state-level policy and concentration of the LGBT population for 2022. After calculating the correlation between these two constructs, the authors identified state-level variation in these measures for each of the 50 states and Washington, DC. With a correlation of 0.58, the findings revealed variation at the state level and indicated that LGBT population concentration and state-level LGBT policy do not necessarily reflect synonymous social phenomena and constitute distinct but complementary measures for use in constructing indices of structural heterosexism and social climate.

Growing polarization in state-level policy environments for LGBT individuals has emerged, with over 500 proposed bills in the 2023 legislative session (American Civil Liberties Union (ACLU), 2023). Alongside the changing policy context, there has been growth in the population of people identifying as LGBT (Jones, 2024), with uneven concentrations across states (Anderson et al., 2021; Cramer et al., 2017; Flores, 2023). A growing body of research has focused on identifying underlying LGBT health disparities, with an emphasis on structural heterosexism (Hatzenbuehler et al., 2024; Homan et al., 2024). Both state-level policies regarding LGBT individuals and measures of concentration of LGBT populations have been included as indicators of heterosexism (e.g. Hatzenbuehler et al., 2024; Prince et al., 2020; Wienke et al., 2021). Although scholars agree that these indicators are related to the well-being of LGBT populations (e.g., Clark et al., 2022; Goldenberg et al., 2020), it is critical to document the share of LGBT individuals exposed to these policies in each state as more hostile policies are enacted.

We analyze recent data from 2022 on the U.S. state policy climates for LGBT individuals and the concentration of the LGBT population. We map the juxtaposition of these two state-level measures. We drew upon two contemporary data sources for this brief: the Household Pulse Survey (HPS) and the Movement Advancement Project (MAP). The HPS is an experimental data product designed by the U.S. Census Bureau to produce estimates at the state level (U.S. Census Bureau, 2022). Although estimates of the LGBT population generated by the HPS are experimental (Anderson et al., 2021), no publicly available dataset currently can provide state-level estimates. The public use National Health Interview Survey (NHIS) file does not include a state indicator and only recently experimented with a gender identity question, and the Behavioral Risk Factor Surveillance System (BRFSS) sexual and gender identity questions are not available in all 50 states and requires the use of multilevel regression with poststratification (Julian et al., 2024). The HPS benchmarks slightly higher estimates of LGBT identification than other national data sources (Julian et al., 2024), but one speculated reason may be differing interview modes (Deng & Watson, 2023). MAP provides an overall measure of LGBT-related laws and policies based on over 50 laws tracked in each state. This measure is presented as a “policy tally” capturing the legal climate for LGBT populations. Our analysis will guide new research on LGBT well-being and state-level indices of heterosexism.

## Background

Although some policies may provide support or protection for vulnerable populations, some policies create hostile or antagonistic environments by targeting those vulnerable populations, resulting in structural stigma, defined as the broader, macro-social forms of stigma that serve to disadvantage already stigmatized populations (Hatzenbuehler & Link, 2014). Evidence shows that social policies are related to many critical outcomes for LGBT populations, including physical well-being (Charlton et al., 2019; Nelson, et al., 2023; Truszczynski et al., 2022), mental health and suicide (Hatzenbuehler, 2011; Raifman et al., 2017), and mortality and birth outcomes (Everett et al., 2022; Hatzenbuehler et al., 2020), as well as indirect measures of well-being such as healthcare access (Tran et al., 2023; White Hughto et al., 2016). LGBT populations in states with protective policies consistently have better well-being outcomes than those residing in states with antagonistic policies (Carpenter et al., 2021; Pearson et al., 2021; Solazzo et al., 2018). To provide an in-depth understanding of the health and well-being of LGBT individuals, researchers need to focus on the potential sources of discrimination and stigma, including the policy environment.

The concentration of LGBT populations within geographic regions has likewise been utilized as a structural indicator of supportive climates because there are more opportunities for interacting with LGBT individuals and community resources. This concentration has often been measured using same-sex couples as the unit of analysis from the decennial Census or American Community Survey (e.g., Gates & Ost, 2004; Julian, 2023) because, until recently, couple-level sex composition measures were the only population-based data available at the state level. Other indicators quantifying LGBT community density have utilized interest group participation data (Taylor et al., 2019) and numbers of Gay-Straight Alliance clubs (Hatzenbuehler et al., 2014a, 2014b). Concentrations of same-sex couples have been frequently used as an indicator of supportive community environments for LGBT individuals (Hatzenbuehler et al., 2014a, 2014b; Wienke et al., 2021).

However, concluding LGBT spatial concentrations solely based on couples omits a large share of the LGBT population (Deng & Watson, 2023), as 63% reported being single (Jones, 2021). Further reliance on same-sex couples as an indicator excludes a large share of individuals who have bisexual identities living with different-sex partners (Jones, 2021; Kühne et al., 2019). The characteristics of LGBT populations are significantly different (e.g., younger, lower education levels, more racially diverse) when considering individuals rather than couples as the unit of analysis (Carpenter & Gates, 2008; Deng & Watson, 2023; Lee et al., 2020). Using couple-level estimates as a proxy for the LGBT population has led researchers to underestimate the impact of geographic characteristics due to the clustering of LGBT individuals in highly diverse areas (Lee et al., 2020).

We move beyond prior studies with our focus on the concentration of the adult LGBT population. Our estimates include a much larger number of individuals—19.6 million estimated LGBT individuals based on the HPS, in contrast to about 2.3 million individuals in same-sex couples in the ACS (Deng & Watson, 2023). Our research aims to map together the state-level concentration of LGBT individuals and the policy environment to show the share of LGBT individuals who are in hostile policy environments and demonstrate how these indicators relate. We build upon prior research on structural factors underlying health and well-being differentials by gender and sexual identity (see Hatzenbuehler et al., 2024 for an overview of indicators on health and structural heterosexism). We contribute by highlighting the proportion of the LGBT population subject to various policy climates and demonstrating the connections between contemporary state-level policy climates and LGBT population concentrations. Our results have implications for future studies of LGBT communities in population measurement, geographic variation, and structural stigma.

## Data and Methods

The U.S. Census Bureau created the Household Pulse Survey (HPS) with 16 other federal agencies to examine the impact of COVID-19 on households (US Census Bureau, 2022b). The Census Bureau collects data in “phases.” Within each phase are weekly intervals, using the Census Bureau’s Master Address File (MAF) as the sampling frame (US Census Bureau, 2022a). In July 2021, the HPS began asking about sexual and gender identity (Anderson et al., 2021). We use data pooled from Week 42 (January 26th to February 7th, 2022) through Week 52 (December 9th to December 19th, 2022) to align with the policy indicators. Aggregating these weeks together, our sample contains 678,222 person-level records. We applied person-level replicate weights to produce estimates for all 50 states and the District of Columbia per Census Bureau recommendations for pooling weeks (U. S. Census Bureau, 2022).

To measure LGBT identification, we used two criteria. For sexual identity, the HPS asked respondents: “Which of the following best represents how you think of yourself?” Response options included: “gay or lesbian,” “straight, that is, not gay or lesbian,” “bisexual,” “something else,” and “don’t know.” Consistent with prior work (Anderson et al., 2021), we considered respondents LGBT if they selected lesbian, gay, or bisexual. We acknowledge that individuals, especially younger adults, may have sexual identities that fall outside a traditional LGB conceptualization and report “something else.” However, we provide a conservative estimate with the understanding that individuals who report “something else” may include non-sexual diverse identities (Julian et al., 2024).

The second criterion focuses on individuals who identify as transgender or report a different gender from their sex assigned at birth (Anderson et al., 2021). The HPS employed a two-step approach for gender identity, aligned with current best practices (Bates et al., 2022). Respondents were asked: "What sex were you assigned at birth on your original birth certificate?" with "male" and "female" response options, then asked: "Do you currently describe yourself as male, female, or transgender?" We based estimates on those who explicitly identified as transgender or reported a sex assigned at birth different from their current gender identity, provided their sex value was not Census Bureau allocated (Julian et al., 2024). LGBT concentration was based on respondents who affirmatively reported an LGB identity or fit within the gender parameters described. All HPS respondents constituted the denominator. We identified 50,568 respondents as LGBT.

Because the age composition of states varies (Kilduff, 2021), and birth year is associated with LGBT identification (Julian et al., 2024), we calculated age-adjusted estimates for each state using the proportion of the U.S. in six birth cohorts (1995–2004, 1985–1994, 1975–1984, 1965–1974, 1955–1964, and 1954 and before) as a weight.

State-level social policy measures were retrieved from the Movement Advancement Project’s indicator based on seven categories: relationship and parental recognition, nondiscrimination, religious exemptions, LGBT youth, healthcare, criminal justice, and identity documents (Movement Advancement Project, 2022). The indicator reflects policies related to sexual identity and gender identification. We find similar results when disaggregating the indicators based on sexual identity or gender identification. The over 50 LGBT-related state-level policies are assigned a point value—positive for protective policies and negative for harmful policies—and these points are totaled into an aggregate policy measure. These policy scores assess laws as written, not necessarily reflecting the implementation or enforcement of laws or resource allocation. We obtained similar results using the 2020 and 2021 (lagged) as 2022 scores.

We estimated the share of the LGBT population who live in states according to the MAP policy scores. We categorized LGBT estimates and policy tallies into terciles and provided shading that indicates low, medium, and high values. Darker shades indicate higher relative population or policy scores, and lighter shades indicate lower relative population or policy scores; these shades overlap to produce a bivariate choropleth map with nine categories.

## Results

**Fig. 1:**
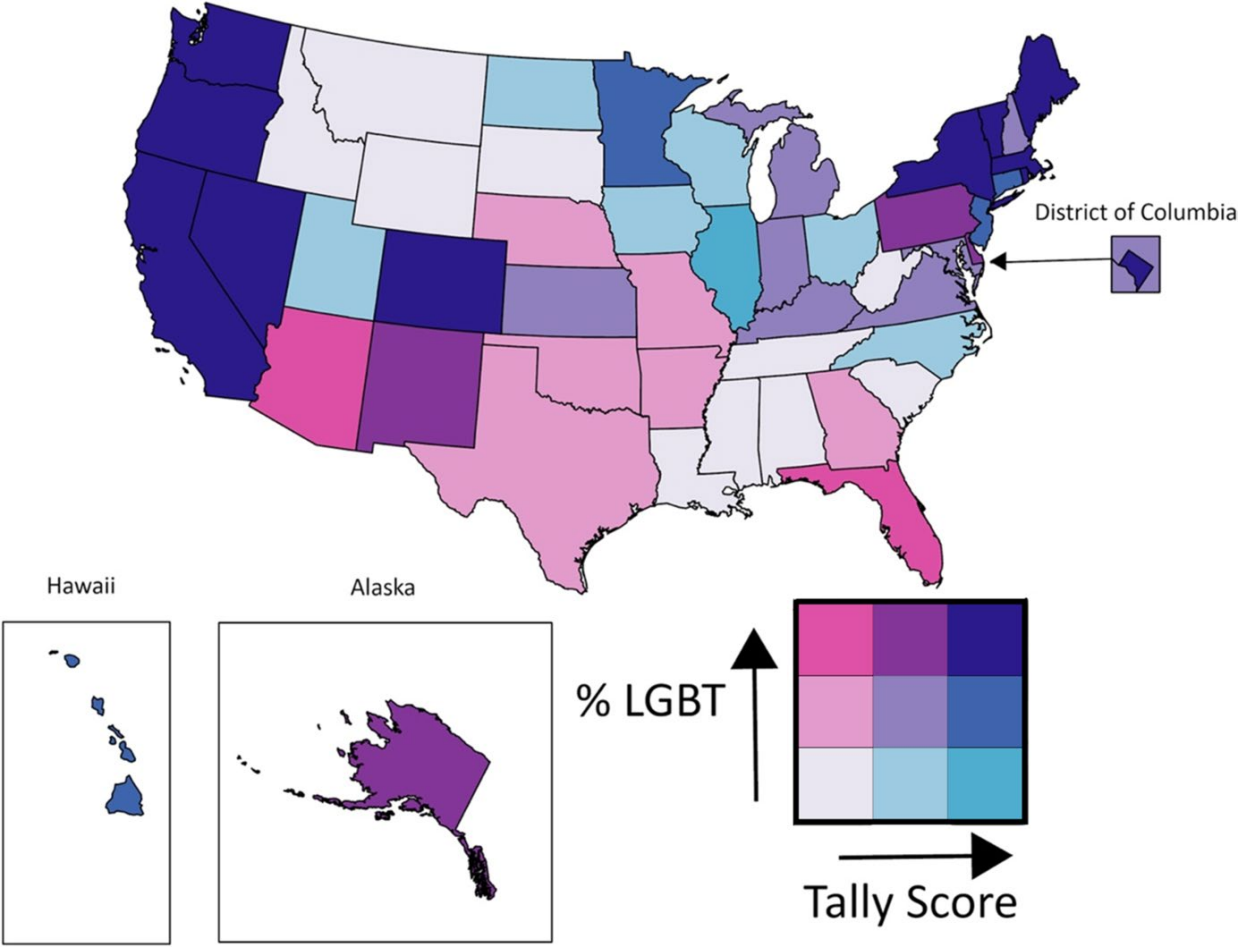
State-Level Policy and LGBT Population Concentration by State

LGBT people represented an overall 8.38% of the United States adult population in 2022, but the proportion of the population that is LGBT varies greatly by state (Table 1). The shading indicates the terciles used to construct Fig. 1. The LGBT concentrations calculated from HPS data range from 4.93% in Mississippi to 16.27% in DC.

**Table 1:** State-level LGBT age-adjusted population concentration by tercile

Tercile	Percent of state population identified as LGBT	States included
Low	4.93%–7.60%	Alabama, Idaho, Illinois, Iowa, Louisiana, Mississippi, Montana, North Carolina, North Dakota, Ohio, South Carolina, South Dakota, Tennessee, Utah, West Virginia, Wisconsin, Wyoming
Medium	7.61%–8.53%	Arkansas, Connecticut, Georgia, Hawaii, Indiana, Kansas, Kentucky, Maryland, Michigan, Minnesota, Missouri, Nebraska, New Hampshire, New Jersey, Oklahoma, Texas, Virginia
High	8.67%–16.75%	Alaska, Arizona, California, Colorado, Delaware, District of Columbia, Florida, Maine, Massachusetts, Nevada, New Mexico, New York, Oregon, Pennsylvania, Rhode Island, Vermont, and Washington

Authors’ Calculations Using the Household Pulse Survey, 2022

State laws relating to LGBT people range from protective to explicitly anti-LGBT in nature. The MAP policy measure for each state includes negative values in eight states (Alabama, Arkansas, Louisiana, Mississippi, Oklahoma, South Carolina, South Dakota, and Tennessee), with an overall range of −8.50 (Alabama) to 41.75 (California) (Table 2). The shading indicates the terciles used in Fig. 1.

**Table 2:** State-level LGBT policy scores by tercile

Tercile	Total policy score	States included
Low	− 8.50–5.25	Alabama, Arizona, Arkansas, Florida, Georgia, Idaho, Louisiana, Mississippi, Missouri, Montana, Nebraska, Oklahoma, South Carolina, South Dakota, Tennessee, Texas, West Virginia, Wyoming
Medium	6.50–29.50	Alaska, Delaware, Indiana, Iowa, Kansas, Kentucky, Michigan, New Hampshire, New Mexico, North Carolina, North Dakota, Ohio, Pennsylvania, Utah, Virginia, Wisconsin
High	30.75–41.75	California, Colorado, Connecticut, District of Columbia, Hawaii, Illinois, Maine, Maryland, Massachusetts, Minnesota, Nevada, New Jersey, New York, Oregon, Rhode Island, Vermont, Washington

Data from Movement Advancement Project Retrieved July 25, 2022

The correlation is 0.58 (p ≤ 0.001) between LGBT population concentration and policy, indicating a positive association but some variability. We find that two-fifths (42.44%) of LGBT adults live in a state with the most supportive policies (top tercile), while one-third (33.26%) live in a state with the least supportive policies (bottom tercile). Specifically, 8.03% of the LGBT population lived in a state with a negative policy score in 2022.

Figure 1 displays each state in a bivariate choropleth map, indicating the terciles of LGBT concentrations and policy scores. Darker shading indicates states with high concentrations of LGBT population, mainly including states on the West Coast and Northeast. The lightest shading is states where the concentration of the LGBT population is low, and the policies are not supportive. These states are in the Southeast (Louisiana, Mississippi, Alabama, Tennessee, South Carolina, and Kentucky) and Northwest (Montana, Idaho, Wyoming, and South Dakota). The states with pink tones have higher concentrations and the most hostile policies (Arizona and Florida). Arizona is in the top LGBT concentration tercile but has a low score on the state policy index, 2.25. The blue tones are states with low relative populations of LGBT individuals but more supportive policies. For example, Illinois is in the lowest tercile for the LGBT population (7.60%) and the uppermost tercile for affirming social policies (35.50).

## Discussion

We aim to illustrate the interplay of state-level concentrations of the LGBT adult population and policy environments. We contribute by providing an analysis of recently collected data and focusing on a broadly defined LGBT population, and not just individuals in same-sex couples. LGBT individuals reside in diverse state-level policy climates; a third (33%) live in the most hostile states, while four out of ten (42%) live in more supportive states. LGBT population and social policies reflect complementary, though not identical, facets of the social climate for LGBT individuals, and it is essential for researchers to understand the variation in these indicators when generating measures of heterosexism, as several states reveal disparities between relative population and social policies, such as Florida, Arizona, and Illinois. These three states occupy opposite terciles in the two measures of interest, representing over 12% of the total LGBT population cumulatively.

The importance of carefully considering the associations between the elements that constitute indicators of structural heterosexism has been similarly displayed in other efforts to generate indicators of structural racism, sexism, and xenophobia (Homan et al., 2024). Our work contributes to a growing understanding of the importance of focusing on place when studying the health and well-being of the LGBT population, as both positive policy climates and community connectedness due to the presence of other LGBT individuals seem to have protective effects on the well-being of LGBT people. We show that a significant proportion of LGBT individuals reside in states with actively hostile policy climates, which is critical to consider in future research on LGBT wellbeing and place.

Although we have illustrated the experiences of LGBT adults in the U.S., our work has a few limitations. First, recent explorations suggest that state-level indicators of LGBT climate are associated with higher response rates to sexual orientation and gender identity-related questions (Zhang et al., 2023). State policy may influence estimates of LGBT population concentration due to the potential fear of disclosing an LGBT identity in a government survey. However, variation in responses to sexual orientation and gender identity items and overall concentrations of LGBT populations cannot be attributed solely to affirming legal climates, as we find there are states with supportive policies that do not show high relative LGBT populations. Second, the sexual and gender identity measures in the HPS are not comprehensive and do not include the terms or language that younger LGBT individuals refer to themselves (e.g., queer or non-binary) (Julian et al., 2024; Suen et al., 2020). Our estimates are based on traditional indicators, and future work should include a more expansive set of measurements of sexual and gender identity.

Third, LGBT adults may be moving in and out of states based on the state-level policy environment, so there may be selection into more supportive states. Unfortunately, the HPS did not include measures of migration patterns. Finally, although the HPS has been critiqued due to the experimental nature of the data and the lower response rates, there are currently no other publicly available population representative datasets that include LGBT identification for all states. As more data become available, we encourage replication of our findings.

We have shown that the interrelation between LGBT population concentration and state-level LGBT policy is complex and reflects significant variation in experienced social climate among the LGBT population. Most notably, we estimate that approximately one-third of all LGBT adults in the United States reside within a state with the most hostile LGBT policies. Researchers will need to take care in accounting for this variation in place in future investigations of LGBT well-being. Efforts to investigate how indicators of heterosexism are associated with critical well-being outcomes for LGBT people in the contemporary context are warranted and may shift as new data become available. This will require integrating existing indicators of structural heterosexism, as well as with emerging measures across geographic units, such as LGBT-based attitudes, hate crimes, politicians’ LGBT identity, interest group strength, and community organizations. New indicators offer opportunities to assess how context and place relate to the health and well-being of the LGBT population, and the widely varying climates in which LGBT people live are important to consider. Efforts to contextualize social climate and the impact of place matters apply to not only research on LGBT populations but all research seeking to identify contextual indicators as they relate to sexism, racism, and xenophobia, as well as the intersection of these forms of discrimination.
